# A new species of *Echinolaophonte* and record of *E. armiger* (Gurney, 1927) (Crustacea, Copepoda, Harpacticoida, Laophontidae) from the Caribbean with a key to species

**DOI:** 10.3897/zookeys.722.14560

**Published:** 2017-12-13

**Authors:** Juan Manuel Fuentes-Reinés, Eduardo Suárez-Morales

**Affiliations:** 1 Universidad del Magdalena, Grupo de Investigación en Biodiversidad y Ecología Aplicada, A. A. 731. Santa Marta, Colombia; 2 El Colegio de la Frontera Sur, Unidad Chetumal, A.P. 424, 77014 Chetumal, Quintana Roo, México

**Keywords:** Benthic copepods, harpacticoids, littoral, taxonomy

## Abstract

A new species of the harpacticoid copepod genus *Echinolaophonte* is described here from specimens obtained during a biological survey of Rodadero Bay, a coastal system in the Colombian Caribbean. This species has been previously recorded as *E. armiger* Gurney, 1927 in different geographic areas (Indian and Pacific Oceans). The Colombian specimens recognized as *E. villabonae* **sp. n.** and true *E. armiger* are deemed as distinct species based on differences in several features of which the shape of the rostrum and the dorsal spinous process on the prosome are among the most distinctive. These and other characters are shared by specimens recorded as *E. armiger* from Caroline Islands and Australia that are now incorporated to the new species. The finding of the true *E. armiger*, previously known only from Egypt, the Texan coast, Brazil, and possibly Bermuda, constitutes the first record of this species in the western Caribbean and a regional range extension. A key to the identification of the 13 known species of the genus is also provided.

## Introduction

The harpacticoid copepod genus *Echinolaophonte* Nicholls, 1941, belonging to the family Laophontidae, was erected to contain several species previously assigned to *Laophonte*. Members of this genus are cosmopolitan, benthic forms (Pesce 2016) that inhabit different marine habitats. *Echinolaophonte* is one of the most diverse genera in the subfamily Laophontinae; it is known to contain 12 valid species (Walter and Huys 2017): *E. armiger* (Gurney, 1927), *E. brevispinosa* (G.O. Sars, 1908), *E. gladiator* (Vervoort, 1964), *E. horrida* (Norman, 1876), *E. hystrix* (Brian, 1928), *E. longantennata* Apostolov, 1990, *E. minuta* Cottarelli & Forniz, 1991, *E. mirabilis* (Gurney, 1927), *E. oshoroensis* Itô, 1969, *E. tetracheir* Mielke, 1981, *E. tropica* Ummerkutty, 1970, and *E. veniliae* Cottarelli, Forniz & Bascherini, 1992. Of these, *E. armiger* has been reported to show a certain degree of variability in the armature of P3-P4EXP and in the urosome ornamentation (Nicholls 1945; Lang 1965; Pesta 1959; Vervoort 1964), therefore, some records have been considered as *species inquirendae* (Wells 2007).

During a survey of the zooplankton community of Rodadero Bay, a shallow coastal system in the Colombian Caribbean, several male and female specimens of harpacticoid copepods of the genus *Echinolaophonte* were obtained. The taxonomic examination of these specimens revealed that some of them were assignable to the strict form of *E. armiger* (Gurney, 1927). Another group of individuals resemble closely *E. armiger* *sensu* Vervoort (1964) and following the opinion by Lee et al. (2006), it was realized that they represent an undescribed species of *Echinolaophonte*. The aim of this paper is to review the status of the records related to *E. armiger* *sensu* Vervoort (1964) and propose a species rank to this taxon by comparing it with its closest congeners based on Colombian specimens. A key to the species currently contained in the genus *Echinolaophonte* is also provided.

## Materials and methods

Biological samples of littoral habitats were obtained from Rodadero Bay, Magdalena, northern Colombia (11°14'10"N, 74°12'06"W) during fieldwork carried out from August 2015 to March 2016, mainly at the inshore areas covered by vegetation (mangrove) and with a bank of oysters. Water salinity, pH, temperature were measured with a multiparameter WTW 350i equipment. Water samples were collected manually using a 25-l bucket at both littoral and limnetic habitats. Samples were then filtered with a plankton net (mesh size = 45 *μ*m) and preserved in 70% ethanol. Copepods were sorted from all the samples and then processed for taxonomical identification including the examination of the whole specimen and dissection of selected appendages. Dissected appendages were mounted on slides with glycerin and sealed with Canada balsam. The specimens were measured in ventral position, from the anterior end of the rostral area to the posterior margin of the caudal rami. Drawings were made with the aid of a camera lucida mounted on an Olympus BX51 compound microscope equipped with Nomarski DIC. Some specimens were prepared for SEM examination with a JEOL LV 5900 microscope at the University of Aguascalientes, Mexico. The process included dehydration of specimens in progressively higher ethanol solutions (60, 70, 80, 96, 100 %), critical point drying, and gold coating following standard methods. The specimens examined were deposited at the Museo de Colecciones Biológicas de la Universidad del Atlántico, Barranquilla-Atlántico, Colombia (**UARC**) and in the Centro de Colecciones Biológicas of the Universidad del Magdalena-Colombia (**CBUMAG**) where they are available for consultation and/or further examination. Morphological terminology follows Huys and Boxshall (1991). The following abbreviations are used in the description: **P1–P6** = first to sixth legs, **EXP** = exopod, **ENP** = endopod.

## Results

### Order Harpacticoida G.O. Sars, 1903

#### Family Laophontidae T. Scott, 1904

##### Genus *Echinolaophonte* Nicholls, 1941

###### Echinolaophonte villabonae sp. n.

Taxon classificationAnimaliaHarpacticoidaLaophontidae

http://zoobank.org/9AA12838-05D6-48FA-88EF-AEDE388D6D8E

**Figure 1.**
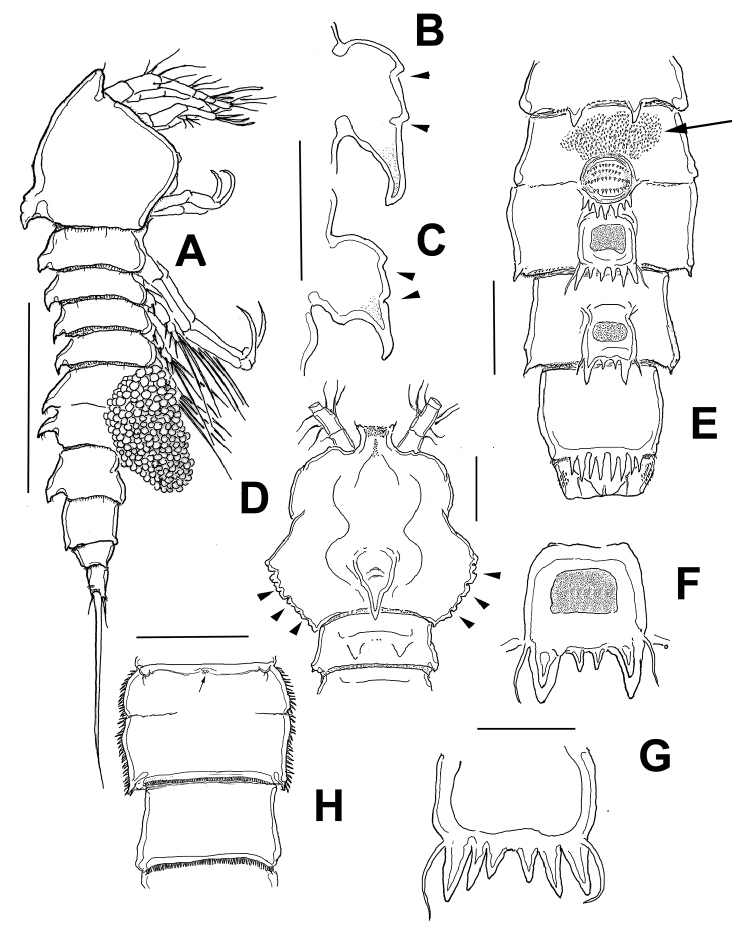
*Echinolaophonte villabonae* sp. n., adult holotype female from Colombia. **A** habitus, lateral view **B** cephalosome dorsal process, lateral view, arrows indicate notches **C** same, another specimen **D** cephalosome showing cuticular indentations on posterolateral margin (arrows), dorsal view **E** genital double, postgenital and preanal somites, dorsal view **F** detail of dorsal process on post-genital double-somite **G** detail of dorsal process, posterior genital double-somite **H** genital double-somite, ventral view showing sixth legs and genital pore (arrowed) on medial anterior margin. Scale bars: 200 μm(**A**); 5 μm (**B–G**).

**Figure 2.**
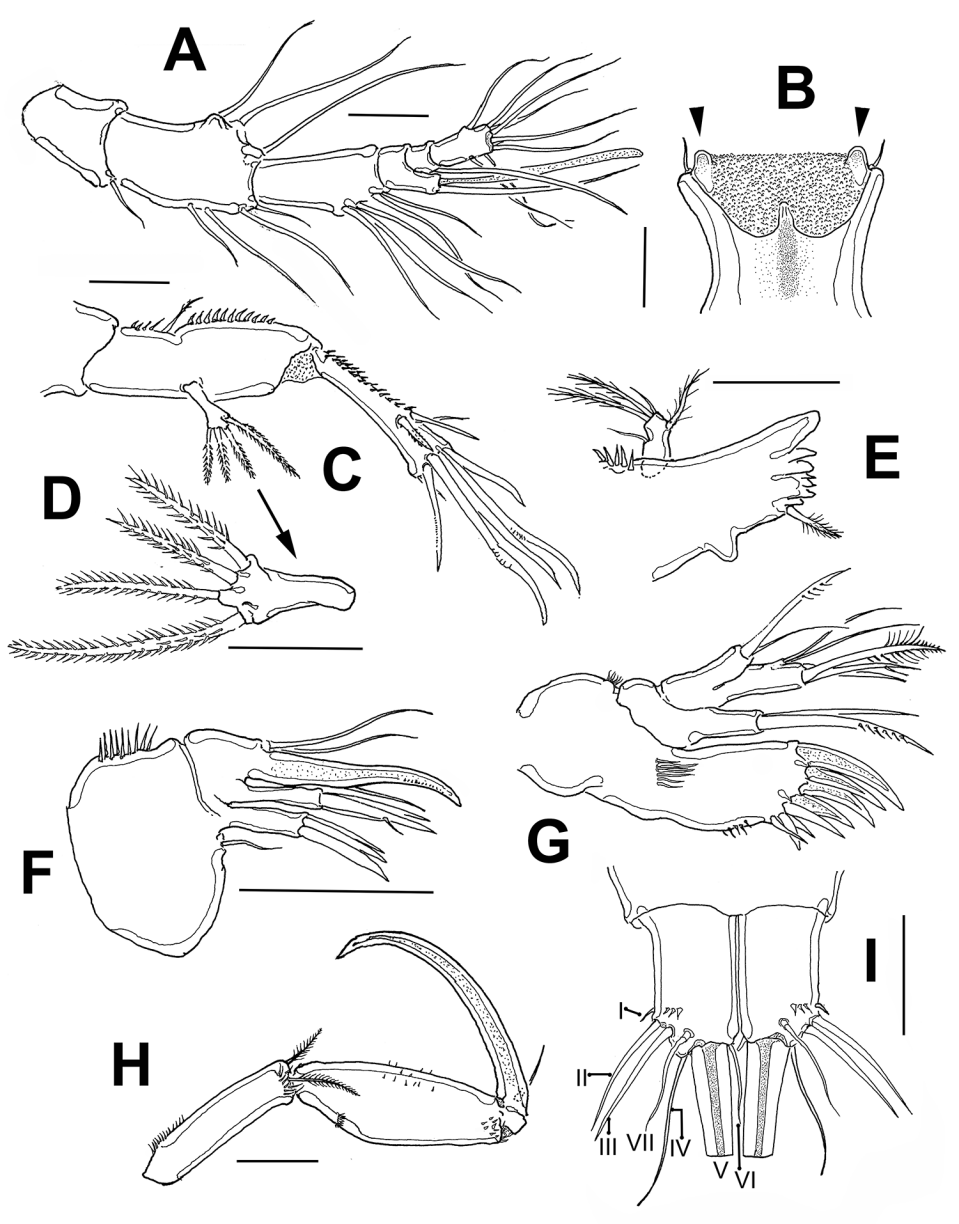
*Echinolaophonte villabonae* sp. n. , adult holotype female from Colombia. **A** antennule **B** rostrum showing surface ornamentation and marginal rounded protuberances (arrowed) **C** antenna **D** antennary exopod, detail **E** mandible **F** maxillule **G** maxilla **H** maxilliped **I** caudal rami showing setae I–VII, dorsal view. Scale bars: 25 μm.

**Figure 3.**
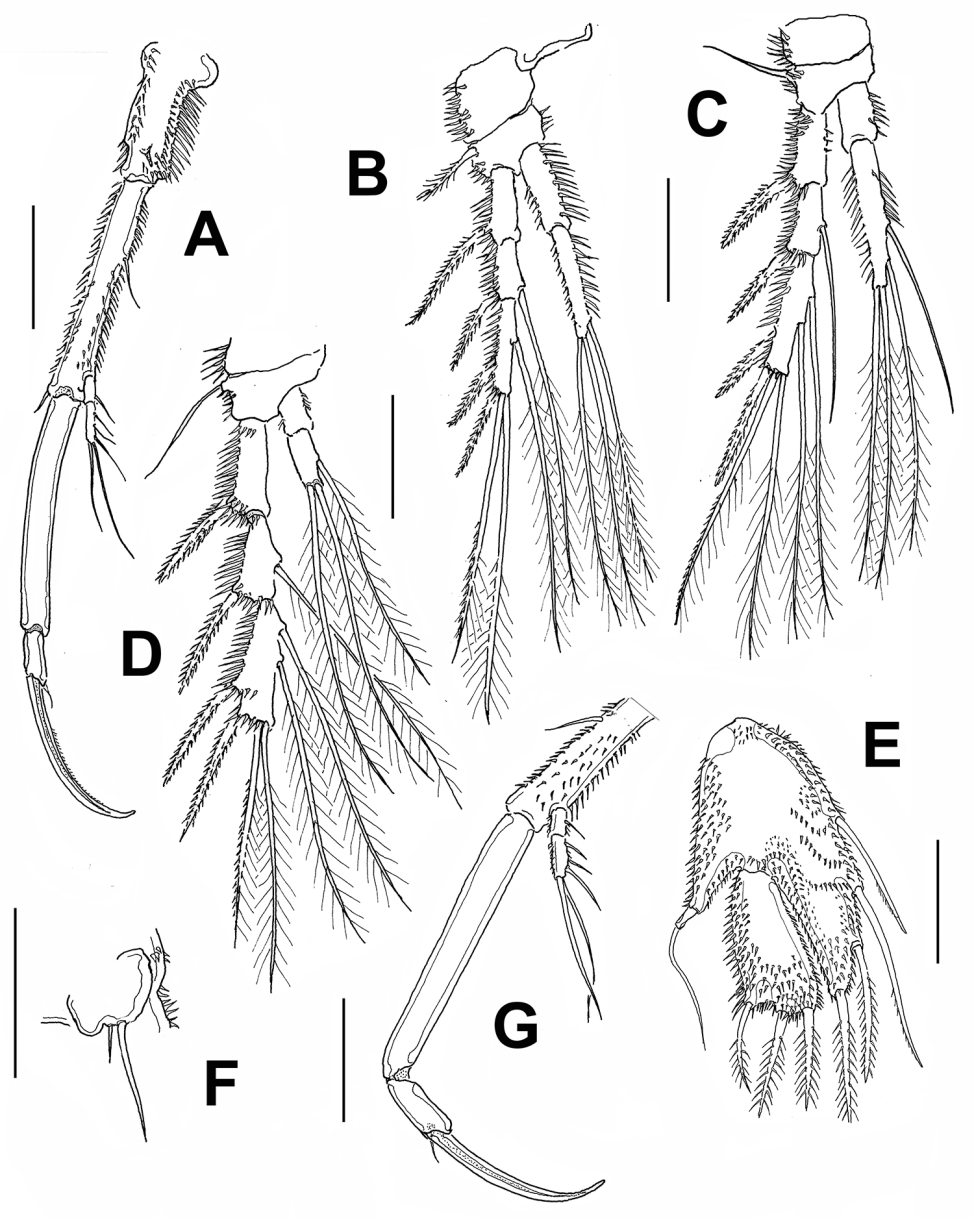
*Echinolaophonte villabonae* sp. n., adult holotype female from Colombia. **A** leg 1 **B** leg 2 **C** leg 3 **D** leg 4 **E** leg 5 **F** leg 6 **G** leg 1, paratype specimen. Scale bars: 50 μm (**A–E, G**); 25 μm (**F**).

**Figure 4.**
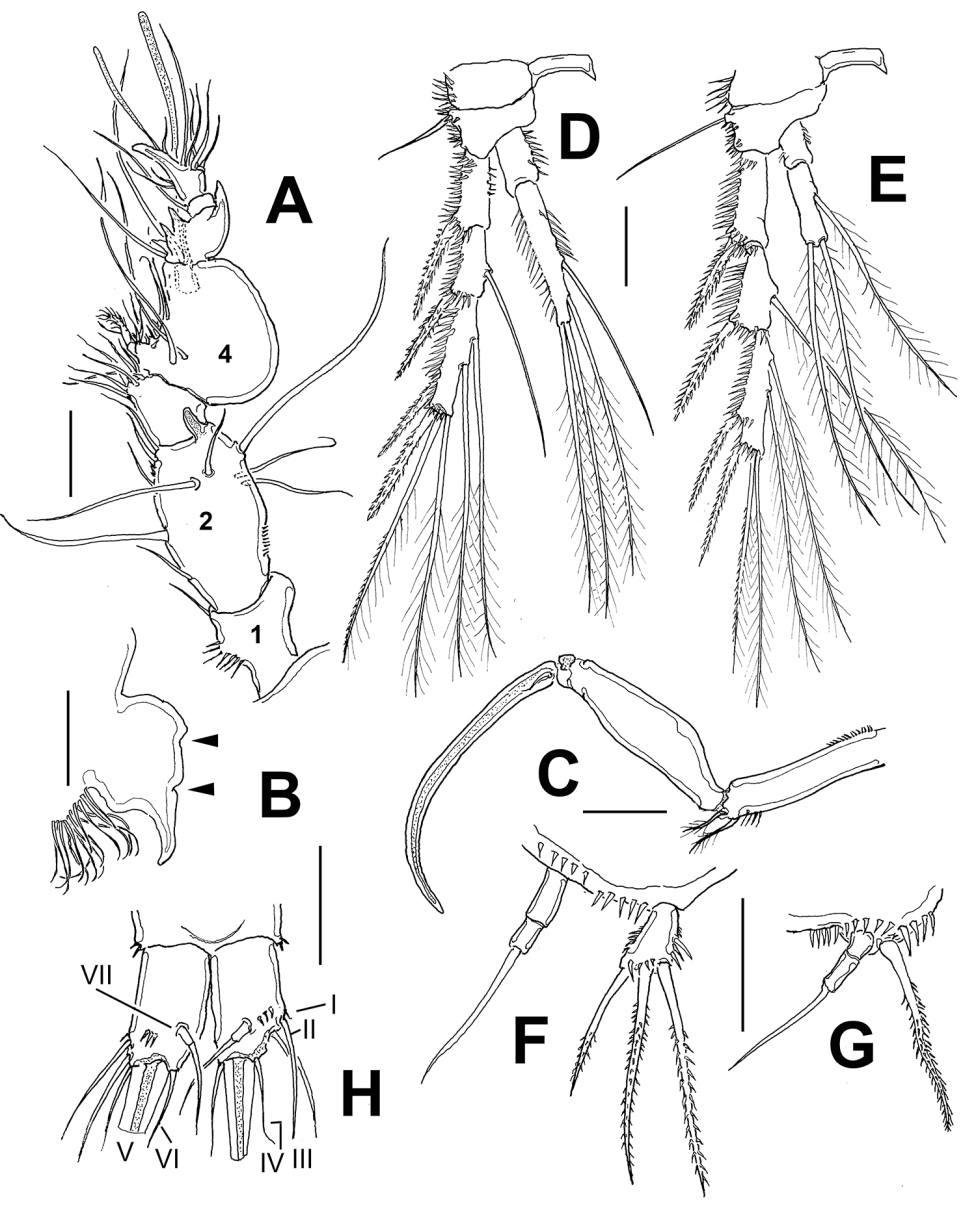
*Echinolaophonte villabonae* sp. n., allotype male from Colombia. **A** antennule **B** cephalosome dorsal process, lateral view, arrows indicate notches **C** maxilliped **D** leg 3 **E** leg 4 **F** leg 5 **G** leg 6 **H** caudal rami showing setae I-VII, dorsal view. Scale bars: 25 μm (**A–G**), 50 μm (**H**).

**Figure 5.**
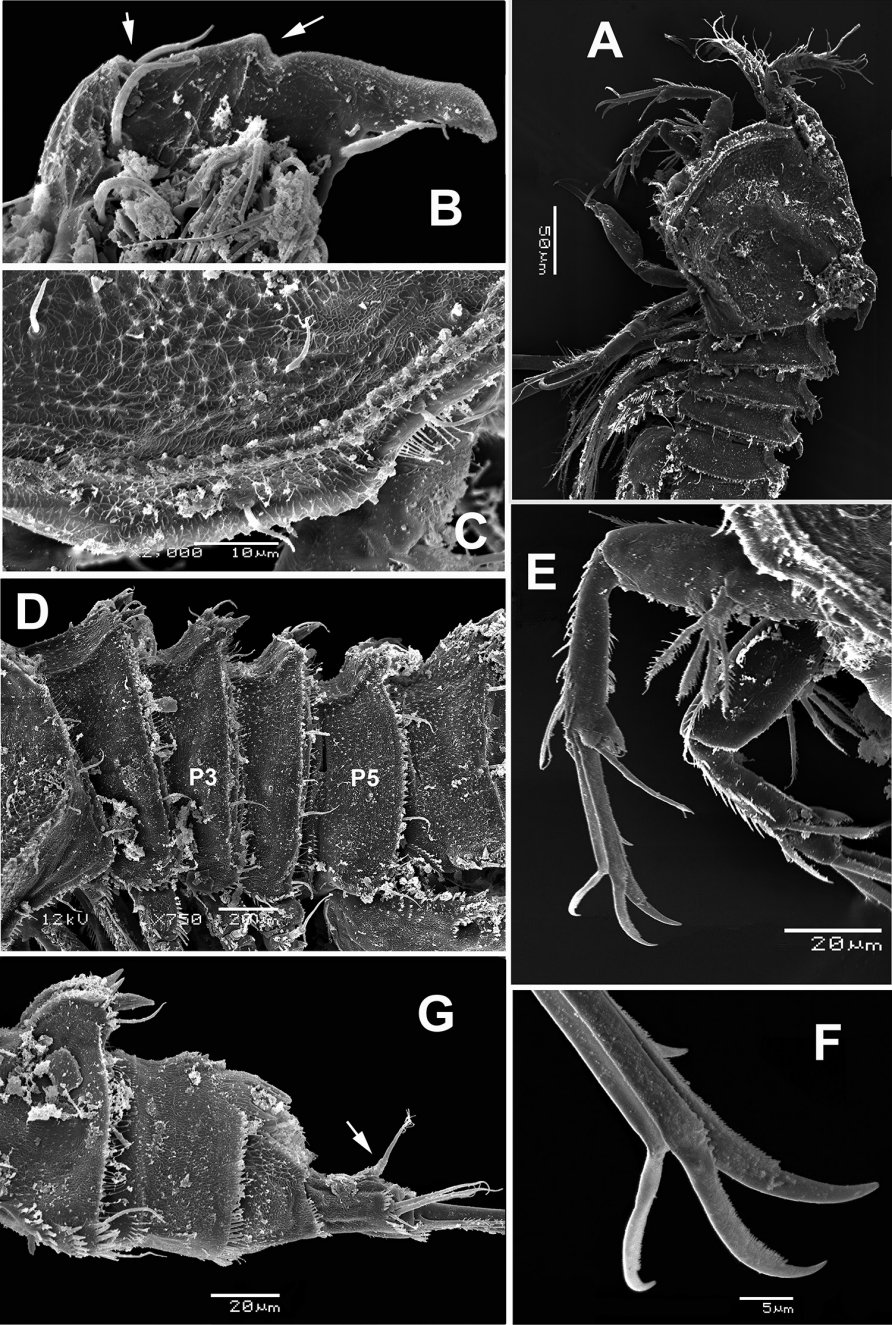
*Echinolaophonte villabonae* sp. n., female from Colombia, SEM-prepared specimen. **A** cephalothorax, lateral view **B** dorsal process on cephalic shield showing distinctive notches (arrowed) **C** detail of cephalic shield marginal ornamentation **D** pedigerous somites 2–5, lateral view **E** antenna **F** detail of distal elements of antenna **G** urosome and caudal rami, lateral view, caudal seta VII arrowed.

**Figure 6.**
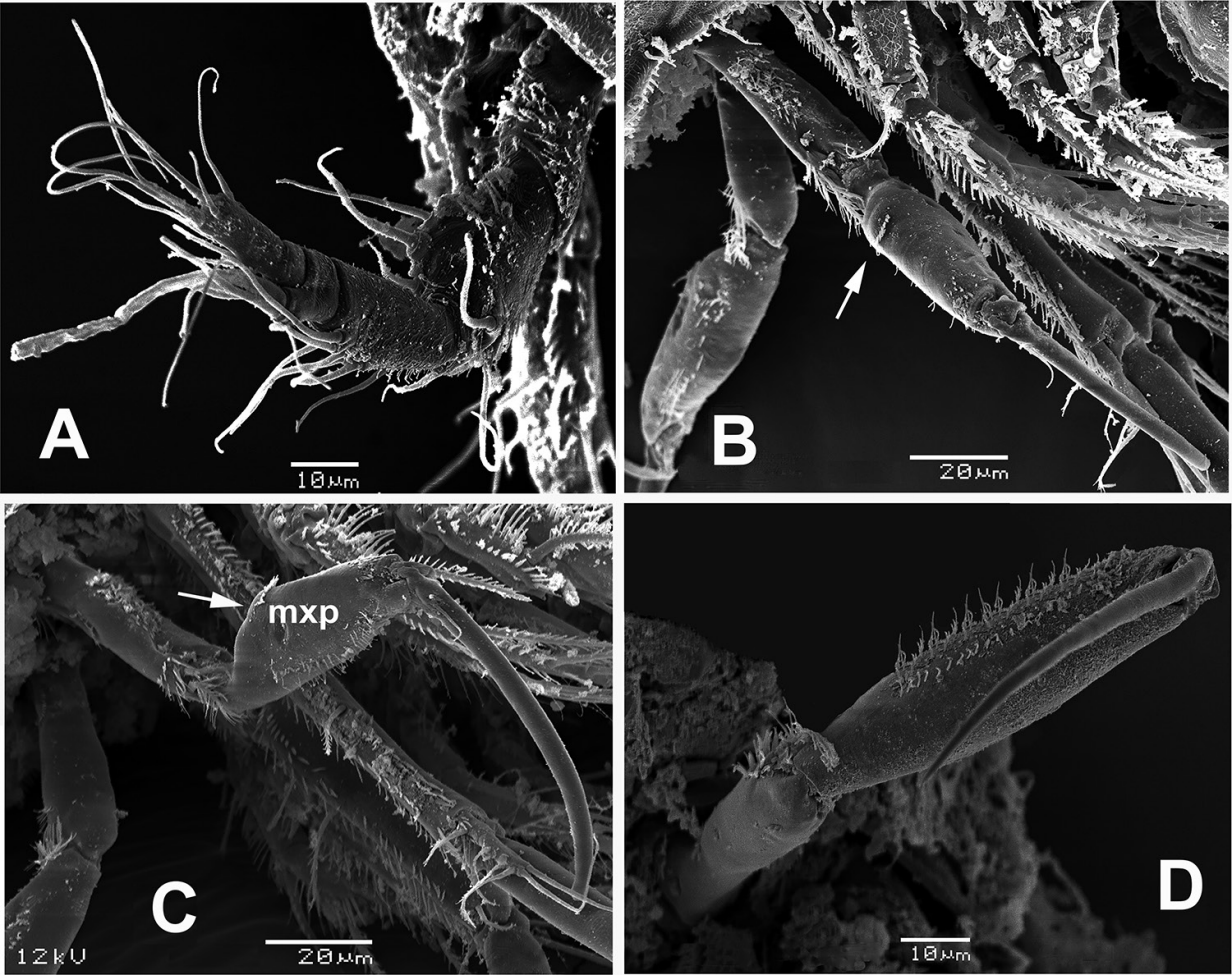
*Echinolaophonte villabonae* sp. n. , female from Colombia, SEM-prepared specimen. **A** antennule **B** maxilliped showing proximal position of spinule comb (arrow); *E. armiger* female from Colombia **C** maxilliped showing medial position of spinule comb **D** male maxilliped.

Figs 1 , 2 , 3 , 4 , 5 , 6A, B

Syn.: Echinolaophonte armiger Nicholls, 1945; Echinolaophonte armiger Vervoort, 1964.

####### Material examined.

Adult female holotype (UARC290M), male allotype (UARC291M), Rodadero Bay, Magdalena, Colombia, coll. J. Fuentes-Reinés, August-June 2016. Paratypes: five females (UARC292M) and two males (UARC293M) from same locality, coll. Juan M. Fuentes-Reinés. Two adult females, two adult males from same locality, date, and collector; specimens dissected, semi-permanent slides (UARC302M–UARC315M). Non-type specimens: two adult females, one adult male in collection of first author (JMFR), one female prepared for SEM analysis, same collection data.

####### Type locality.

Rodadero Bay, Magdalena, northern Colombia (11°14'10"N, 74°12'06"W).

####### Diagnosis.

Body cylindrical, rostrum wide, subrectangular, medially flat, posterior margin of cephalic shield with strong dorsal spiniform which possess two notches. Female antennule six-segmented; male antennule subchirocer, seven-segmented. Antenna three-segmented, EXP one-segmented with four strong setae, inner longest. Mandible ENP and EXP reduced, with three and one short pinnate setae, respectively. Maxillule with well-developed arthrite and eight distal elements; EXP one-segmented, with two apical setae. Maxilliped three-segmented, endopodal claw with single seta. P1-P4ENP and EXP being two and three-segmented, respectively, but P1EXP two-segmented. P1ENP 7.5 times as long as wide. Female and male P5 with long setophore and apical seta, female P5 EXP and ENP with three and four setae, respectively, male P5EXP with three setae. Female and male caudal rami with seven setae.

####### Description.

*Female*. Habitus as in Figure 1A. Body cylindrical in dorsal view, prosome gradually tapering anteriorly. Total body length measured from anterior margin of rostrum to posterior margin of caudal rami ranging from 560 to 616μm (average = 586.7 μm, *n* = 11; holotype: 588 μm). Strong dorsal spiniform process present at median posterior margin of cephalic shield (Figs 1A, 5A) and reaching middle of second pedigerous somite. Process with two distinctive notches on posterior margin, distal end represented by curved point (Figs 1B–C, 5B).

Cephalothorax with smooth posterior margin; lateral posterior corners of cephalic shield produced into triangular expansions (Fig. 1D) and intricate cuticular ornamentation (Fig. 5B, C). Urosome five-segmented, in lateral view urosomites 3-5 with strong expansion, cuticular surface ornamented with minute denticles, posterior margins spinulate (Figs 1A, 5D). Genital double-somite (Fig. 1G, H) with transverse, shallow suture on ventral surface, indicating original intersomite segmentation; dorsal surface with ornamentation as in figure 1E, including a field of minute spinules on proximal dorsal surface (arrowed). Genital pore inconspicuous, located medially on anteriormost end of somite (Fig. 1H). Some specimens carrying single egg sac ventrally; egg mass set close to ventral surface of genital and postgenital somites (Fig. 1A). Postgenital somite relatively narrow, with large dorsal spiniform process in lateral view (Fig. 5G); with medial expansions visible in dorsal view (Fig. 1F); surface with cuticular reticulation. Posterior margin ornamented with row of short setules and ventral rows of spinules (Fig. 5G). Succeeding preanal somite lacking large dorsal spiniform process but with posterodorsal expansions visible in dorsal view (Fig. 1E); distal margin of somite and posterolateral surface furnished with spinules and reticulate cuticular surface (Figs 1E, 5G). Anal somite tapering posteriorly, with row of minute spinules on posterior margins (Fig. 5G).

Caudal rami 1.4–1.9 times longer than wide; each ramus with seven setae: setae I–III bare, first one shortest, setae IV and V pinnate, fused at base, seta VI approximately half-length of seta IV, seta VII articulated (Fig. 2I, arrow in Fig. 5G). Rostrum wide, subrectangular, medially flat (Fig. 2B), with rounded protuberance on each end of distal margin (arrows in Fig. 2B), and pair of sensilla. Antennule (Fig. 6A) and antenna (Figs 2A, C, D, 5E, F) as in *E. armiger* *sensu* Vervoort (1964) (Lee et al. 2016).

Mandible (Fig. 2E) gnathal blade with several multicuspid teeth plus pinnate dorsal seta, dorsal margin with subdistal rounded protuberance. Palp with small basal seta; endopod and exopod reduced, represented by expansions armed with three and one short pinnate setae, respectively.

Maxillule (Fig. 2G). Precoxal arthrite with eight distal spines/setae. Subdistal row of small spinules on inner margin of arthrite. Coxa with cylindrical endite bearing stout smooth seta and curved, distally uniserially pinnate spine. Basal endite cylindrical, armed with two naked setae and pinnate spine. Endopod incorporated in basis, forming small peduncle with two naked slender setae. Exopod one-segmented, with two apical setae.

Maxilla (Fig. 2F) comprising syncoxa with two endites furnished with spinules along outer margin plus short inner distal seta. Coxal endites each with three pinnate spines. Allobasis transformed into strong, slightly curved, distally pinnate claw. Endopod represented by two setae.

Maxilliped (Figs 2H, 6B) represented by cylindrical syncoxa armed with two distal plumose setae with rows of slender hair-like elements at insertion of setae. Basis nearly twice as thick as syncoxa, widest at midlength, ornamented with comb of spinules on proximal 1/3 (arrowed in Fig. 6B), medial field of scattered spinules, and distal rows of slender setae. Endopod forming long curved claw with short setulated seta at base.

P1 (Fig. 3A, G). Coxa ornamented with rows of spinules on inner and outer margins, as figured. Protuberant tube pore on subdistal medial position. Basis with single slender seta on proximal 1/3 of segment, segment furnished with spinules on inner and outer margins. EXP short, two-segmented, EXP1 with one outer seta, EXP2 with five setae, two apical. ENP1 7.5 times as long as wide, ENP2 short, 2.1 long as wide with strong, denticulate apical claw and small, naked seta at base. EXP short, ¼ the length of ENP1.

P2 (Fig. 3B). Coxa and basis ornamented as figured. Basis with stout outer basipodal seta. EXP three-segmented, longer than endopod; EXP1 lacking inner seta, EXP 2 with inner seta. ENP1 lacking inner seta, ENP2 four times as long as wide with one inner and two apical elements. ENP reaching halflength of EXP3.

P3 (Fig. 3C). Coxa and basis ornamented as figured. Basis with outer basal seta. EXP three-segmented. EXP1 without inner seta, EXP2 with inner seta. ENP two-segmented, ENP1 lacking inner seta, second segment almost five times as long as wide with two inner and two apical elements. ENP barely reaching beyond distal margin of EXP2.

P4 (Fig. 3D) Coxa and basis ornamented as figured. Basis with outer basipodal seta. Exopodal ramus three-segmented. EXP1 without inner seta, EXP2 with inner seta. ENP two-segmented, ENP1 lacking inner seta, ENP2 segment almost four times as long as wide with one inner and two apical elements. ENP short, not reaching distal margin of EXP1. Spine/ setal formula of P2–P4 as follows:

**Table:** 

	**Basis**	**Exopod**	**Endopod**
P2	1-0	I-0;I-1;II,2,2	0-0;2,1
P3	1-0	I-0;I-1;I,I-2,2	0-0;2,2
P4	1-0	I-0;I-1;I,I-2,2	0-0;2,1

P5 (Fig. 3E). EXP and baseoendopod covered with small spinules as figured. Baseoendopod with long outer setophore armed with single apical seta. Endopodal lobe not reaching distal margin of exopod, with one apical and three lateral, pinnate setae. EXP elongate, twice as long as wide, with three pinnate setae.

P6 (Fig. 3F). Represented by two setae, a short inner one, and a longer outer one.

*Male.* Habitus resembling that of female but somewhat smaller. Total body length measured from anterior margin of rostrum to posterior margin of caudal rami ranging from 476 to 508 μm (average = 478 μm, *n* = 7; holotype: 476 μm).Cephalosome with strong dorsal spiniform at median posterior margin of cephalic shield as in female (Fig. 4B). Antennule (Fig. 4A) subchirocer, seven-segmented, geniculation between fourth and fifth segments. First segment with row of spinules, second segment with small subdistal knob. Fourth segment swollen; fifth segment with spiniform processes. Seventh segment with triangular expansion on distal half.

Antenna, mandible, maxillule, and maxilla as in female. Maxilliped as in female (Fig. 4C) except for narrower basis and relatively longer claw. P1 and P2 as in female (not illustrated). P3 (Fig. 4D) as in female except for outer spines on EXP1-3, slightly stronger than in female.

P4 (Fig. 4E) as in female except for EXP3 and outer spine on EXP1-2, narrower and slightly stronger, respectively, than in female.

P5 (Fig. 4F) clearly separated at base. Baseoendopod with long setophore bearing apical seta. Exopod slightly longer than maximum width, with three pinnate setae and row of spinules on anterior surface.

P6 (Fig. 4G) represented by one bipinnate inner and one naked outer seta. Outer seta arising from setophore.

Caudal rami 1.5–1.6 times as long as wide (Fig. 4H).

####### Etymology.

The species is named after Dr. Silvia Lucía Villabona-González, for her intense research on the zooplankton communities of Colombia and for her legacy and leadership of new generations of planktologists in this country.

####### Remarks.

The genus *Echinolaophonte* was divided by Lang (1965) into two lineages, the first one is characterized by its possession of 3, 3, 2 outer spines on P2-P4 EXP3, respectively, and the male P3ENP is transformed, with an apophysis in the second segment. The second lineage shows 2, 3, 2 outer spines on P2–P4 EXP3, respectively, and the male P3ENP is not modified; it lacks an apophysis on the second segment, as in the female. Most species can be accommodated in these two lineages but *E. minuta* has a mixture of such characters and could fit in both groups (spinal formula 2, 3, 2, and male P3ENP2 with apophysis); a similar situation is true for *E. tetracheir* Mielke, 1981. Two species do not match the characters of any of these linages: *E. gladiator* (Vervoort, 1964) and *E. mirabilis* (Gurney, 1927). They have an outer spine formula of 2, 2, 2 and 3, 3, 3, respectively; males of both species remain unknown. The new species, *E. villabonae* shares the same set of lineage characters with *E. gladiator*; therefore, this division should be reevaluated or expanded.

The redescription of *E. armiger* based on type material from the Suez Canal, Egypt supported the notion that only a few of the previous records of *E. armiger* *sensu* Gurney (1927) actually correspond to this species (Lee et al. 2006). One of these uncertain records of *E. armiger* is that provided by Vervoort (1964); these specimens differ from the “true *E. armiger* (Gurney, 1927)” in several characters as previously outlined by Lee et al. (2006) who suggested that Vervoort’s (1964) specimens represent an undescribed species. They also recognized that Nicholl’s (1945) Australian record of *E. armiger* is assignable to *E. armiger* *sensu* Vervoort (1964). However, the specific identity of these two groups of specimens was not established because of the difficulty to observe and confirm key characters in the preserved specimens and by the low number of individuals available for examination. The recent finding of a large number of specimens of E. cf. armiger in plankton samples from Rodadero Bay, Colombia, which are almost identical to those reported as *E. armiger* *sensu* Vervoort, 1964 from Port Denison (Australia) (Nicholls 1945) and Caroline Islands (Vervoort 1964), motivated a wider analysis of this problem in order to determine and establish the true identity of these specimens. As a result, a new species of *Echinolaophonte* is erected based on the Colombian specimens after comparing them with Gurney’s true *E. armiger* (Gurney, 1927).

**Figure 7.**
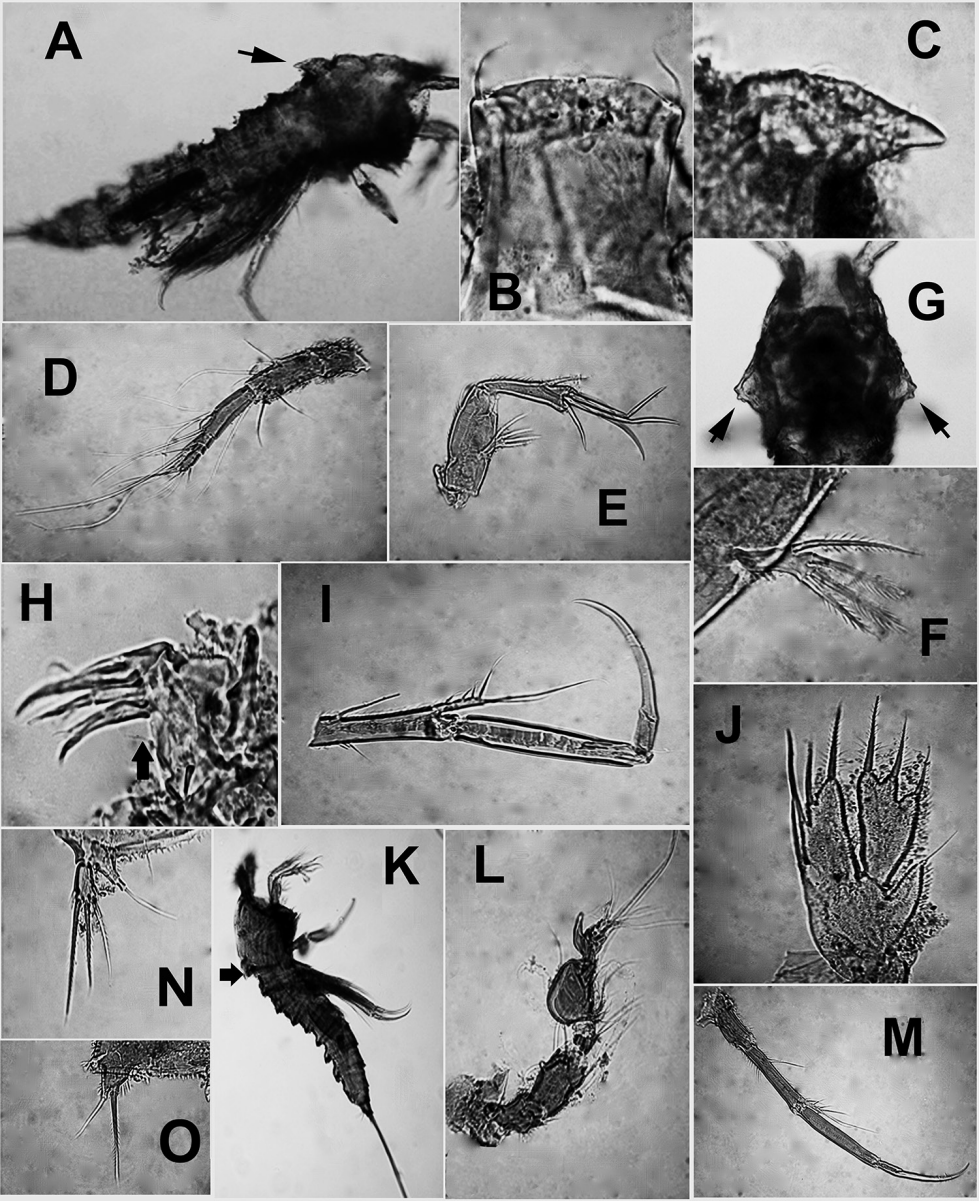
**A–O** *Echinolaophonte armiger* (Gurney) from Colombia, unscaled digital micrographs. Adult female (body length = 644 μm). **A** habitus, lateral view showing dorsal process on cephalic shield (arrow) **B** rostrum showing flat anterior surface and depressed distal margins **C** process on cephalic shield, lateral view **D** antennule **E** antenna **F** detail of antennary exopod **G** cephalosome showing weak development of posterolateral margins (arrows), dorsal view **H** maxillule showing slender inner seta on syncoxa (arrowed) **I** leg1 **J** leg 5; adult male (body length 560 μm) **K** habitus, lateral view showing smooth dorsal process on cephalosome **L** antennule **M** leg1 **N** leg 5 **O** leg 6.

The new species, *Echinolaophonte villabonae* sp. n. can be distinguished from *E. armiger* Gurney, 1927 by several characters:1) the rostrum has two protuberances in its anterior margin in *E. villabonae* (Fig. 2B) whereas it is flat in *E. armiger* (Fig. 7B, Lee et al. 2006, fig. 1A) 2) the maxilla bears a small inner coxal seta in *E. villabonae* (Fig. 2F) whereas this seta is absent in *E. armiger* from the type locality (Lee et al. 2006, fig. 2F), but it was observed in the Colombian material (arrow in Fig. 7H; 3) the maxillipedal base has a spinule comb in the proximal 1/3 in *E. villabonae* (Fig. 2H) whereas this comb is medial or subdistal in *E. armiger* (Fig. 6C, Lee et al. 2006, fig. 2C); in both species male maxillipeds are slenderer than in the female (Figs 4C, 6D); 4) the shape of the dorsal spinous process has two distinctive notches on the dorsal margin in *E. villabonae* (visible in lateral view, Figs 1B, C, 5B) whereas in *E. armiger* the same structure is smooth (Fig. 7A, C, Lee et al. 2006, fig. 1B); 5) the length/width ratio of P1ENP1 is 7–7.5 in *E. villabonae* (Vervoort, 1964, fig. 143a, as *Onychocamptus armiger*, Fig. 3A, G) vs. 5.6–5.8 in *E. armiger* (fig. 7I, Lee et al. 2006, fig. 3A); 6) the length/width ratio of P2-P4ENP2 is 6.25, 6.0,and 2.5, respectively in *E. villabonae* sp. n. vs. 3.9, 5.8, and 3.8 in *E. armiger*; 7) in *E. armiger* the distal margin of P4ENP reaches beyond the point of insertion of the outer spine of the elongate P4EXP1(Lee et al. 2006, fig. 4B) whereas in *E. villabonae* the endopod is clearly shorter and does not reach this level (Fig. 3D); 8) the number of setal elements on P3EXP3 also differs between these two species: it has 6 in *E. villabonae* (Vervoort 1964, fig. 143C, as *O. armiger*; Fig. 3C) vs. 7 in *E. armiger* (Lee et al. 2006, fig. 4A); 9) the setophore of the P5 outer basal seta is relatively longer in *E. villabonae* (Fig. 3E) than in *E. armiger* (Lee et al. 2006, fig. 3C); 10) the dorsal ornamentation of the urosome is clearly stronger in *E. villabonae* (Figs 1E–G, 5G) than in *E. armiger* (Lee et al. 2006, figs 1B, 5B). The male of *E. villabonae* sp. n. shows some additional differences with respect to *E. armiger*: 1) the antennule is 7-segmented in *E. villabonae* (Fig. 4A) vs. 8-segmented in *E. armiger* (Lee et al. 2006, fig. 6B); 2) the caudal rami ratio is 1.5–1.6 in *E. villabonae* (Fig. 4H) vs. 1.4 in *E. armiger* (Lee et al. 2006, fig. 6A). The erection of this new taxon and the comparisons with the other related records of this species allows us to allocate all previous records of *Echinolaophonte armiger* *sensu* Vervoort (1964) in the Indian and Pacific Ocean as belonging to the new species, *E. villabonae*.

The new species most closely resembles *E. gladiator* Vervoort, 1964 and *E. tropica* Ummerkutty, 1970. They share an identical armature formula of P1–P4. The female fifth leg armature, with 3 and 4 setal elements on the fifth leg EXP and ENP, respectively also resembles the pattern found in *E. gladiator* (Vervoort 1964, fig. 145e), and *E. tropica* (Ummerkutty 1970, fig. 3O; Wells and Rao 1987, fig.145a). However, *E. villabonae* can be separated from these species when comparing the length/width ratio of P1ENP which is 4.4 in *E. tropica* (Wells and Rao 1987, fig. 144c), 5.8 in *E. gladiator* (Vervoort 1964, fig. 145a) and 7.5in *E. villabonae* (Fig. 3A). Also, the length of P4ENP with respect to the elongate P4EXP1 is different in these species: in *E. tropica* (Wells and Rao 1987: fig. 144f) and *E. gladiator* (Vervoort 1964, fig. 145d), the endopod reaches the point of insertion of the outer spine of the first exopodal segment, whereas in *E. villabonae* the endopod is clearly shorter and does not reach this level (Fig. 4E). The structure of the dorsal spinous process reveals additional differences: in *E. villabonae* (Figs 1B, C, 5B; Vervoort 1964: fig. 142b) and *E. gladiator* (Vervoort 1964: fig. 144c) a conspicuous process is present, being much longer and with two notches in the former whereas in *E. gladiator* the structure is clearly smaller and lacks such processes; this process is absent in *E. tropica* (Ummerkutty 1970: fig. 3B; Wells and Rao 1987: fig. 142d). We consider that the evidence presented justifies the separation of a new species of *Echinolaophonte*; it comprises previous records of *E. armiger* by Nicholls (1945) and Vervoort (1964) and raises the number of known nominal species to 13.

####### Distribution and habitat.

This species is known to be associated mainly with coral reef areas. It has been reported (as *E. armiger*) from the reef area at Port Denison, Australia (Nicholls 1945) and also from the Ifaluk Atoll, Caroline Islands in the Pacific (Vervoort 1964). In Colombia this species was found in the littoral zone of the Rodadero Bay in an area covered by mangrove with a small adjacent bank of oysters at a depth of 0.70 m and a water temperature ranging between 30 and 32 °C, salinity 36.1 psu, pH 8.3. Its finding in the Northwestern Atlantic Ocean suggests that it is widely distributed in tropical latitudes.

###### Echinolaophonte armiger

Taxon classificationAnimaliaHarpacticoidaLaophontidae

(Gurney, 1927)

Figs 6C–D , 7

Syn. Laophonte armiger Gurney, 1927: 554–556, fig. 159; Willey 1930: 108–109, figs 65–67; Carvalho 1952: 159–160, Pl. II, figs 68–71.Onychocamptus armiger Lang, 1948: 1423–1424, Abb. 571(12), 580.

####### Material examined.

One dissected adult female (CBUMAG:MEI:0003), two adult males and four adult females, ethanol-preserved, vial (CBUMAG:MEI:0002; CBUMAG:MEI:0001); Colombia, Magdalena, Rodadero Bay, 11°14'N, 74°12'W, August, 2016; coll. J.M. Fuentes-Reinés. One male, one female prepared for SEM analysis.

####### Description.

*Female*. Habitus as in Figure 7A. Body cylindrical in dorsal view, prosome gradually tapering anteriorly. Total body length measured from anterior margin of rostrum to posterior margin of caudal rami ranging from 616 to 644 μm (average = 624.4 μm, *n* = 5). Rostrum conical in lateral view, trapezoid with flat anterior margin in dorsal view (Fig. 7B). Strong, dorsal spinous process present at median posterior margin of cephalosome reaching midlength of second pedigerous somite; process smooth in lateral view (arrow in Fig. 7A, C). Cephalosome with lateral posterior corners produced into triangular expansions (arrows in Fig. 7G) with weak cuticular incisions.

Antennule (Fig. 7D) 6-segmented, with long aesthetasc on fourth segment. Antenna (Fig. 7E, F) three-segmented, comprising coxa, allobasis, one-segmented endopod and one-segmented exopod, antennal exopod one-segmented with four well developed, pinnate setae (two lateral, two apical).

Mandible, maxillule, maxilla, and maxilliped (Fig. 6C) as in syntype specimens of *E. armiger* (Lee et al., 2006), except for seta on maxillary coxa (arrow in Fig. 7H).

P1 (Fig. 7I) with ENP and EXP two-segmented, ENP1 length/width ratio = 5.6, ENP2 ratio = 2.8, with denticulate claw and small, naked seta at base. EXP short. EXP1 with unipinnate spine. EXP2 with three unipinnate spines and two geniculate setae. P2-P4 as described by Lee et al. (2006).

P5 (Fig. 7J) with EXP and baseoendopod separate; each covered with spinules. Baseoendopod with short setophore bearing basal seta. Endopodal lobe armed with four setae, exopodal lobe with three. P6 represented by one inner small and one outer longer seta. Caudal rami length/width ratio = 1.4.

*Male*. Habitus resembling that of female (Fig. 7K). Total body length ranging from 532 to 588 μm (average = 560 μm, *n* = 3).Cephalosome with strong dorsal spiniform medial process as in female (arrowed in Fig. 6K). Antennule (Fig. 7L) subchirocer, eight-segmented, with geniculation between segments 5 and 6. First segment with row of spinules, second segment with small subdistal knob. Segment 5 swollen, segment 6 with spiniform processes. Maxilliped with relatively narrower basis and longer terminal claw than in female (Fig. 6D).

P1 (Fig. 7M) and P2 as in female; P3-P4 as in female, except for outer and distal spines of exopod which are slightly thicker than in female. P5 (Fig. 7N) fused medially, defined at base. Baseoendopod with short setophore bearing long outer basal seta, endopodal lobe obsolete. Exopod narrow, armed with three pinnate setae and spinules on anterior surface. P6 (Fig. 7O) represented by subquadrate plate armed with bipinnate inner and naked outer seta. Caudal rami length/width ratio = 1.4.

####### Variability.

One male was observed to have three setal elements on the antennal exopod instead of the usual pattern of four.

####### Remarks.

The present record, from the Caribbean coast of Colombia, is the fifth of *E. armiger* *sensu* Gurney, 1927 worldwide. In the surveyed area this species coexists with the closely related *E. villabonae*, which is locally more abundant. Lee et al. (2006) redescribed *E. armiger* from syntypic specimens from the Suez Canal and complemented their report with specimens from the Gulf of Mexico (Texan Coast, USA); the species is characterized by: 1) the smooth apical margin of the rostrum, 2) the simple dorsal spinous process on the cephalosome, 3) the armature formula of the swimming legs, 4) the short ENP2 of P2-P4, 5) the unmodified P3 endopod in male, 6) the slightly modified exopods of P3 and P4 in male, 7) the short setophore of the P5 outer basal seta, 8) short caudal ramus (only 1.4 times longer than wide), 9) the shape of dorsal spiny processes in the prosome and urosome and 10) the shape of pseudoperculum. Most of these distinctive traits were observed in the Colombian specimens. However, subtle differences were detected in our specimens from Colombia, thus allowing an expansion of the morphological range of this species: 1) a maxillary coxal seta is present in the Colombian specimens (Fig. 7H) whereas it is absent in the Texan material (Lee et al. 2006: fig. 2F), 2) the P1ENP1 length/width ratio. It is 5.6 in the Colombian specimen (Fig. 7I) whereas in the Texas specimens the figure is slightly higher: 5.8 (Lee et al. 2006: fig. 3A).

####### Distribution.

Suez Canal, Egypt (type locality), the Texan coast (USA) (Lee et al. 2006), Brazil (Carvalho 1952), northern Colombia (present data), and possibly Bermuda (Willey 1930).

### Key to the known species of *Echinolaophonte*

**Table:** 

1	Spinal formula of outer spines on P2-P4 EXP3 = 332 or 232	**5**
–	Spinal formula of outer spines on P2-P4 EXP 3 = 222 or 333	**2**
2	Spinal formula of outer spines on P2-P4 EXP 3 = 222	**3**
–	Spinal formula of outer spines on P2-P4 EXP 3 = 333	***E. mirabilis* (Gurney, 1927)**
3	Dorsal spiniform process present on cephalic shield, P2ENP reaching halflength of EXP3, length/width ratio of P1 ENP between 4.5 and 5.8	**4**
–	Dorsal spiniform process on cephalothorax absent, P2ENP reaching inner seta of P2EXP2, length/width ratio of P1 ENP = 4.4	***E. tropica* Ummerkutty, 1970**
4	Dorsal spiniform process with two distinctive notches on posterior margin, second segment of antennule without particular features, length width ratio of P1 ENP = 7.5.	***E. villabonae* sp.n.**
–	Dorsal spiniform process smooth, lacking notches on posterior margin, second segment of antennule with strong, outwardly directed hook, length/width ratio of P1 ENP = 5.8	***E. gladiator* (Vervoort, 1964)**
5	Spinal formula of P2-P4 = 232, male P3 ENP with or without apophysis	**6**
–	Spinal formula of P2-P4= 332, male P3 ENP with or without apophysis	**8**
6	Baseoendopod of female P5 with 2 inner setae; EXP3 P2 as long as segments 1-2 combined. EXP2-3 P2 lacking inner setae	***E. minuta* Cottarelli & Forniz, 1991**
–	Baseoendopod of female P5 with single inner seta; EXP1-3 P2 subequal in length. EXP2-3 P2 with inner setae	**7**
7	P4 ENP shorter than EXP1, rostrum reverse trapezoid, small, body size 580 µm (female), 490 µm (male)	***E. hystrix* (Brian, 1928)**
–	P4 ENP longer than EXP1, rostrum rectangular, body size 618-650 µm (female), 565 µm (male)	***E. armiger* (Gurney, 1927)**
8	Female P5ENP with four setae, maxillule with endopod	**9**
–	Female P5ENP with two setae, maxillule lacking endopod	***E. veniliae* Cottarelli, Forniz & Bascherin, 1992**
9	Caudal rami length/width ratio 4 times as long as wide, male P3 ENP lacking apophysis	***E. tetracheir* Mielke, 1981**
–	Caudal rami length/width ratio between 3.1 and3.4 times as long as wide, EXP2 P2 with two setal elements, inner seta and outer spine; EXP3 P2 with inner seta	**10**
–	Caudal rami length/width ratio ranging between 2.0 and 2.2 times as long as wide, EXP2 P2 with one setal element, inner seta absent, EXP3 P2 lacking inner seta	**11**
10	P1 EXP reaching halflength of ENP1, P2ENP1 not reaching distal end of EXP1, rostrum bilobed at tip, male P3EXP2-3 inner setae long	***E. brevispinosa* Sars, 1908**
–	P1 EXP relatively short, reaching ¼ the length ENP1, P2ENP1 reaching distal end of EXP1, rostrum rounded at tip, male P3EXP2-3 inner setae short	***E. horrida* (Norman, 1876)**
11	P2 ENP2 with medial distal seta spiniform, clearly shorter than adjacent two distal setae, half the length of bearing segment. P1 ENP1 lacking inner seta	***E. longantennata* Apostolov, 1990**
–	P2 ENP2 with medial distal seta setiform, as long as adjacent 2 setae. P1 ENP1 with inner seta	***E. oshoroensis* Itô, 1969**

## Supplementary Material

XML Treatment for Echinolaophonte villabonae

XML Treatment for Echinolaophonte armiger
